# Investigation into the Structural, Chemical and High Mechanical Reforms in B_4_C with Graphene Composite Material Substitution for Potential Shielding Frame Applications

**DOI:** 10.3390/molecules26071921

**Published:** 2021-03-29

**Authors:** Ibrahim M. Alarifi

**Affiliations:** 1 Department of Mechanical and Industrial Engineering, College of Engineering, Majmaah University, Al-Majmaah, Riyadh 11952, Saudi Arabia; i.alarifi@mu.edu.sa; Tel.: +966-50-222-2828; 2 Engineering and Applied Science Research Center, Majmaah University, Al-Majmaah, Riyadh 11952, Saudi Arabia

**Keywords:** boron carbide, nanoparticles, graphene, composite materials, shielding

## Abstract

In this work, boron carbide and graphene nanoparticle composite material (B_4_C–G) was investigated using an experimental approach. The composite material prepared with the two-step stir casting method showed significant hardness and high melting point attributes. Scanning electron microscopy (SEM), along with energy dispersive X-ray spectroscopy (EDS) analysis, indicated 83.65%, 17.32%, and 97.00% of boron carbide + 0% graphene nanoparticles chemical compositions for the C-atom, Al-atom, and B_4_C in the compound studied, respectively. The physical properties of all samples’ B_4_C–G like density and melting point were 2.4 g/cm^3^ density and 2450 °C, respectively, while the grain size of B_4_C–G was in the range of 0.8 ± 0.2 µm. XRD, FTIR, and Raman spectroscopic analysis was also performed to investigate the chemical compositions of the B_4_C–G composite. The molding press composite machine was a fabrication procedure that resulted in the formation of outstanding materials by utilizing the sintering process, including heating and pressing the materials. For mechanical properties, high fracture toughness and tensile strength of B_4_C–G composites were analyzed according to ASTM standard designs. The detailed analysis has shown that with 6% graphene content in B_4_C, the composite material portrays a high strength of 134 MPa and outstanding hardness properties. Based on these findings, it is suggested that the composite materials studied exhibit novel features suitable for use in the application of shielding frames.

## 1. Introduction

**Figure 1:**
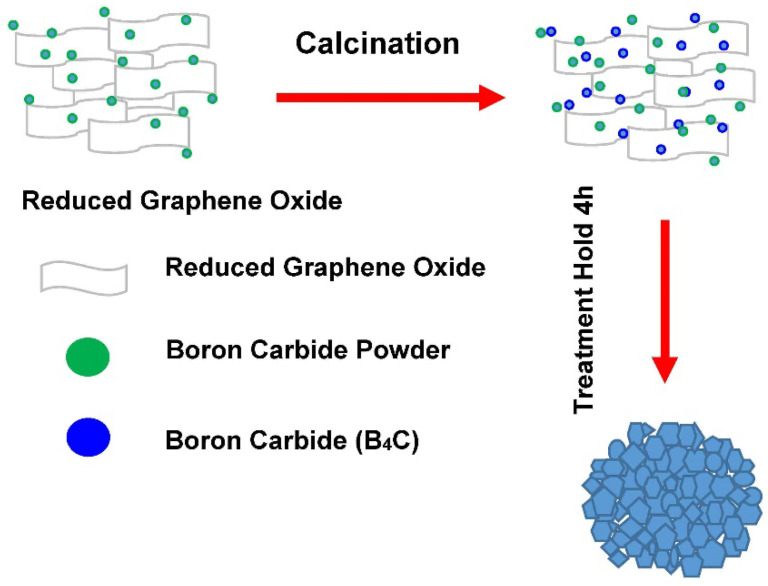
The formation process of the B_4_C–G composite.

Boron carbide (B_4_C) is one of the most significant and widely used materials in the shield engineering field for use in shielding reactors. The industry requires those functional materials which can sustain extreme conditions and perform dependably for the shielding frame. Several studies have been conducted to investigate the chemical and mechanical characteristics, and the hardness of B_4_C material. Subramanian et al. [1] studied B_4_C and suggest its use in the shielding industry due to its low density and notable hardness. The mechanical properties and microstructure of B_4_C–G platelet composites were also investigated. The highest toughness mechanisms for the increased fracture toughness are crack deflection, crack branching and crack bridging [2]. In another study, Domnich et al. [3] provided a comprehensive review covering the concept of structural and chemical variations in boron carbide. Fine-grained B_4_C nanoparticles were synthesized through a carbon thermal process for successful photo-electro catalytic hydrogen generation activity in water reduction [4]. In another approach, B_4_C nanoparticles were prepared from ball milling, and their potential use as a novel agent in T-cell-guided boron neutron capture therapy, were investigated [5]. Chen et al. [6] conducted a microscopic and spectroscopic investigation of synthesized B4C nanomaterial and have confirmed its unique chemical and physical properties. The formation of B_4_C nanoparticles was also reported by a solid-state thermal reaction in a conventional high-temperature furnace reactor [7]. Crystalline B_4_C manifested the high-density twin structure. The computational studies on B_4_C oxidation suggest B_4_C degradation can be manipulated to directly influence global bundle degradation (see Figure 1) [8]. In addition, Kovziridze et al. [9] investigated the influence of Ti and Zr doping on B_4_C and found they improved the mechanical strength of the B_4_C composite.

Furthermore, the percentage of chemical components in the experimental material’s chemical composition is in the range of 0.2–0.4%, and the purity of boron carbide is within the range 97–98% according to standard ASTM C750-18 [10]. In this work, the graphene substitution strength of B4C was improved by graphene because graphene is a widely accepted material that can be used to enhance the host material’s mechanical and chemical attributes and synthesis, determined by XRD GO-B4C and rGO-B4C [11]. The B_4_C–G nanoparticles were studied in detail and exhibited enhanced strength and chemical bonding. The Raman spectra and XRD results for boron carbide reveal that the carbon atoms in all samples were preferentially substituted by taking boron atoms, and the atoms’ effective distances at the end of the B4 units was shorter than that of C–B–C atom chains, which can cancel out the increased bonding length affected by B substitution [12]. Boron and silicon carbide composite materials are significant for nuclear shielding applications, which are essential for neutron shielding and gamma [13]. The achievement in a high-speed planetary ball mill is boron carbide in a nanostructured formation state, in a 10–15 µm grain size [14].

This research study’s novelty is that it refers to B4C, which is among the most promising shielding material and in high demand for novel applications in the shielding industry. By incorporating the newest technology, high shielding proficiency can lead the way to numerous new applications. The B_4_C composite studied has shown strong suitability for shielding frames due to its improved hardness, and its high melting (2450 °C) and boiling points (3500 °C). Furthermore, graphene-based composite materials are of high interest to researchers due to their considerably good physical, microstructure, chemical, mechanical, and morphological properties. The challenge is to show the effect of the nanoparticles during the preparation of the studied materials. In the mechanical formation process, the mixing of graphene into the B_4_C was undertaken in a steel molding press to obtain composite shielding materials that could be used in a shielding frame. All of the properties of the composite materials are discussed in this work.

## 2. Experimental Work

### 2.1. Materials

In this work, B_4_C in powder form was used with boron and carbon, whose percentages fell within the range 78–81% and 17–22%, respectively, according to the Sigma Aldrich material specifications. The B_4_C specimens were synthesized in the experimental laboratory facilities provided by Majmaah University. A sufficient and pure grade of fixed B_4_C, typical element size: 0.2 µm concentration >98%) and formless boron (Graphene. 0%, 2%, 4%, and 6% concentration; typical element size: 0.3 µm) powders were synthesized by roll milling to make B_4_C–G nanoparticles. Based on ASTM standards, a fabricated pressing machine was then used to investigate the B_4_C–G mechanical properties. The crystal structure of the B_4_C used was rhombohedral with an average grain size of about 0.8 ± 0.2 µm. Multilayer graphene was added to the boron carbide by constant percentages from 0–0.6% according to the main specifications of the ASTM WK56764 [15].

### 2.2. Methods

#### 2.2.1. Heat Treatment

The calcination process of the B_4_C–G prepared nanoparticles was performed in an air furnace at graphene absorption concentrations of 0%, 0.2%, 0.4%, and 0.6%. The process was completed in two hours, with the maximum temperature of the furnace raised to 160 °C. In the next step, the carbonization was achieved by raising the temperature to 1200 °C in nitrogen with a 10 Psi pressure for 2 h (Model-OTF-1200X-High temperature vacuum tube Furnace Manufacturer Richmond, USA). In this process, 0% oxygen content was used to avoid engaging the materials in a leaching process, as that would have affected the result, its differentiation between the heat treatment process of the solubility, and the chemical composition.

#### 2.2.2. Preparation and Solidification

**Figure 2:**
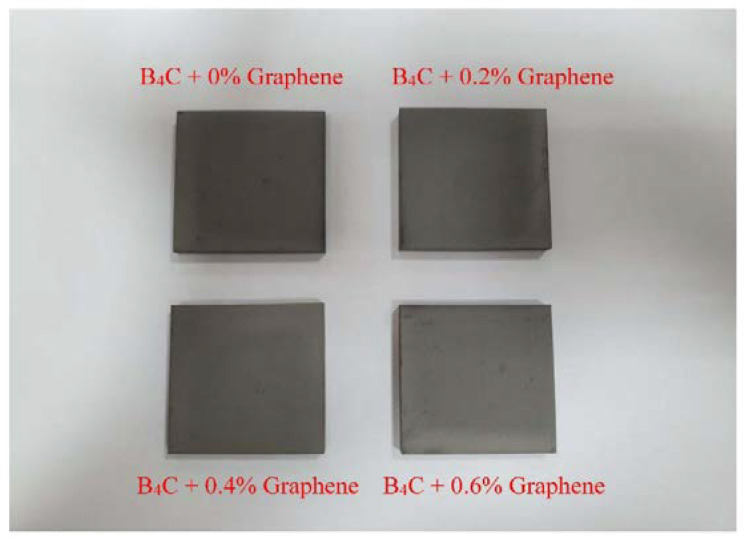
The B_4_C–G samples of composite materials.

The effect of B_4_C particles on the mechanical and physical properties of four base composite samples was examined using B_4_C of 0, 0.2, 0.4, and 0.6 wt % of graphene. All samples were prepared and tested at the laboratories of the College of Science at Majmaah University. A two-step stir casting method was used to make the composite samples. The composite was squeezed into solidification for 5 min with a squeezing pressure of 89 MPa, using a hydraulic press (YKC Series H-type Fin Press Line). The cast samples were later heated to 300 °C for 3 h for stress release, and then allowed to cool in the air. The stir casting and squeezing systems utilized are presented in Figure 2.

#### 2.2.3. SEM + EDS Test

The scanning electron microscopy (SEM) with energy dispersive spectroscopy (EDS) measurements were performed using a field emission scanning electron microscope (FE-SEM)—JEOL Model (JSM-6500). MEYEB specimens’ fracture surfaces were glazed in gold (8 nm thick), which enabled scanning by using an ion beam sputter coater Hummer VII, and maximum resolution images (below 2 µm) were obtained. 16-kV beam energy was used on the specimens (roughly 2.5 mm thick) in the internal chamber with continuous vacuuming. SEM spectroscopy was used to measure the nano- or micro-particles size in the morphology of the material’s chemical components, and to measure the powders’ micro-grain structure size [15]. SEM images show the boron carbide B_4_C with different nanoparticle content. The SEM images have a visibility feature to define the high porosity level of all the boron carbide B_4_C/micro-size graphene samples. This analysis has fully observed low and dense sintering at low temperatures during the forming of boron carbide B_4_C and graphene particles. 

#### 2.2.4. XRD Test 

The X-ray energies were detected with high accuracy and improved resolution. In order to minimize the noise level and recognize closely packed X-ray peaks, the accepted practice is to specify the EDS resolution at Mn-kα (crystal, energy). EDS was performed on the SEM, including AMTEK EDAX, Model (Octane), with 40 mm^2^ detectors used for measuring. The detector has a 115 eV at Mn-Kα resolution. The X-ray peaks were collected from the pre-specified points on the sample to view its appearance with the maximum 13 kV SEM electron beams. X-ray diffraction and particle size analyses has been carried out for the B_4_C powder in detail. 

#### 2.2.5. Raman Spectrum Test 

The Raman spectroscopy technique is an excellent tool for examining microelement structures. The Raman analysis was undertaken using a Kaiser Model (Holo-Pro) spectrograph built-in a (CCD) camera from Princeton Instruments. It functioned at 967 nm by using a 35 mW power supply as the excitation source. The laser light was focused on the fibers and then back dispersed at 180 °C and recorded by an f/1.5 collection lens. The crystal orientation intensity peaks were obtained according to the average from 10 samples. The lower and higher frequency regions were observed in terms of the Raman bands ratio [14]. The structure and the chemical asymmetry affected the Raman intensities and the expected low energy in the B–O chain structure. The Raman spectroscopy band could be obtained by enhancing B–C bonds with the replacement of B with C in the icosahedra. 

#### 2.2.6. FTIR Test 

Fourier transform infra-red (FTIR) spectroscopy is a beneficial tool for defining chemical interaction through the annealing of B_4_C–G samples. The measurement started at around 36 °C and was ramped up to 420 °C in a platinum pan. FTIR analysis was concluded using a Bruker spectrometer. Infrared spectra were detected in the wavenumber range of 500–4000 cm^−1^.

#### 2.2.7. Tensile Test 

Standard specimens were prepared according to ASTM D3039-17, and the computer control universal testing device model MTS (E45) manufactured in the US was utilized, which has a tensile speed rate of 0.2 mm/min at room temperature. The four samples were tested for tensile and hardness for B_4_C–G. The chemical composition of the aluminum wire used, and the percentage composition breakdown between B4C and graphene, are demonstrated in Table 1. The molding press procedure resulted in the formation of superior materials using the sintering process, which includes heating and pressing the materials. In this work, the mechanical properties revealed high fracture toughness and high strength for the materials analyzed according to ASTM standard designs. The composite material studied exhibited properties of improved high strength, density, and outstanding hardness. 

**Figure 3:**
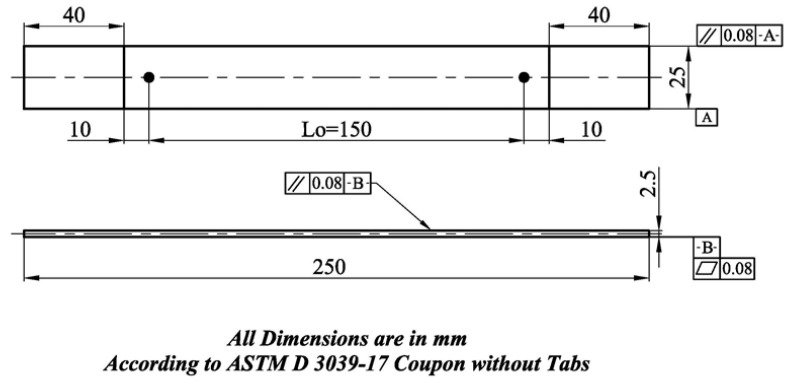
Main dimensions of the tensile test specimen according to ASTM D3039-17 [16].

The thickness of the tensile test specimen significantly affected the selection of the sample configuration, and the tensile test sample’s main dimensions. The standard methods have been classified as the configuration of tensile test specimen according to the application’s thickness, such as sheet, plate, pipe, and composite structures. Figure 3 illustrates the main dimensions of the tension test specimen made from composite materials, B_4_C–G, according to standard ASTM D3039-17 [16]. The gauge length of the tensile specimen was Lo = 150 mm, thickness t = 2.5 mm, and specimen width, w = 25 mm. This increased the accuracy of measuring the tensile specimen’s cross-section area, and the average area was estimated based on three areas across the gauge to calculate an average = WXT. The surface of tap length was equal to 40 mm from both ends of the tensile test specimen. The distance between the tap length and the beginning of the gauge length was equal to 10 mm from both ends (see Figure 3). Furthermore, the tap length’s friction surface was increased by using an emery cloth to minimize the sliding between the universal tensile testing machine’s grip and the tensile test specimen during the test [17]. The tensile test specimen experiment was conducted at a crosshead speed equal to 2 mm/min and environmental room temperature 23 ± 3 °C based on standard ASTM D3039-17 [16].

#### 2.2.8. Fracture Toughness Test

The fracture toughness test is a simple technique used to define the fracture toughness of brittle materials compared with traditional methods of measuring a specimen’s toughness. B_4_C material is one of the hardest and most brittle materials that suffers from brittle fracture behavior under certain operating conditions. Therefore, the indentation fracture toughness test was the most suitable method for predicting the fracture toughness, and the KIC for boron carbide material-based hardness test according to standard ASTM C1327-15 [18]. The main hardness test specimen dimensions were fixed according to the specimen’s thickness; for instance, the hardness sample’s length must be 10 times the thickness. The thickness of the hardness specimen must be at least 0.50 mm. Suitable grinding and finishing were carried out before exposing the samples (250 mm length × 25 mm height, and 2.5 mm width) to the hardness test. The hardness test was conducted on a Micro Brunel’s Vickers hardness device, Qualitest manufactured in Canada, using a weight of 31.25 Kg for 12 s. The hardness was verified by taking the average of four hardness readings for each sample.

Moreover, a variation in specimen thickness does not affect the hardness value. It increases the accuracy for measuring the hardness values, and the standard test recommended is to use at least five to ten specimens in each hardness test. The Vickers hardness test method was used to calculate the hardness of boron carbide according to Equation (1) as follows:

(1) $$Hv=0.0018544{\left(\frac{P}{d^{2}}\right)}^{2}$$

where *P* is the applied load, and *d* is the average length of the two indentation area diagonals for the Vickers indenter. The Vickers’ indentation area and the length of the diagonals’ indentation area was d1 and d2. The configuration of the indentation area plays an essential role in determining the hardness values. The acceptable and the unacceptable regions according to the Vickers indenter can accurately portray the prediction of a hardness test according to ASTM C1327-15 [18].

In this work, the indentation fracture toughness of boron carbide was calculated according to Equation (2) [19]:

(2) $$Kic=0.0018P{\left(\frac{E}{c^{3}Hv}\right)}^{\frac{1}{2}}=0.0264a{\left(\frac{EP}{c^{3}}\right)}^{\frac{1}{2}}$$

where *P* is the load used, *E* is Young’s modulus or modulus of elasticity from the tensile test, the diagonal length of the indentation area is 2*a*, the Vickers hardness, *Hv*, and crack length is 2*c*.

## 3. Results and Discussion 

### 3.1. SEM Microstructure Analysis 

**Figure 4:**
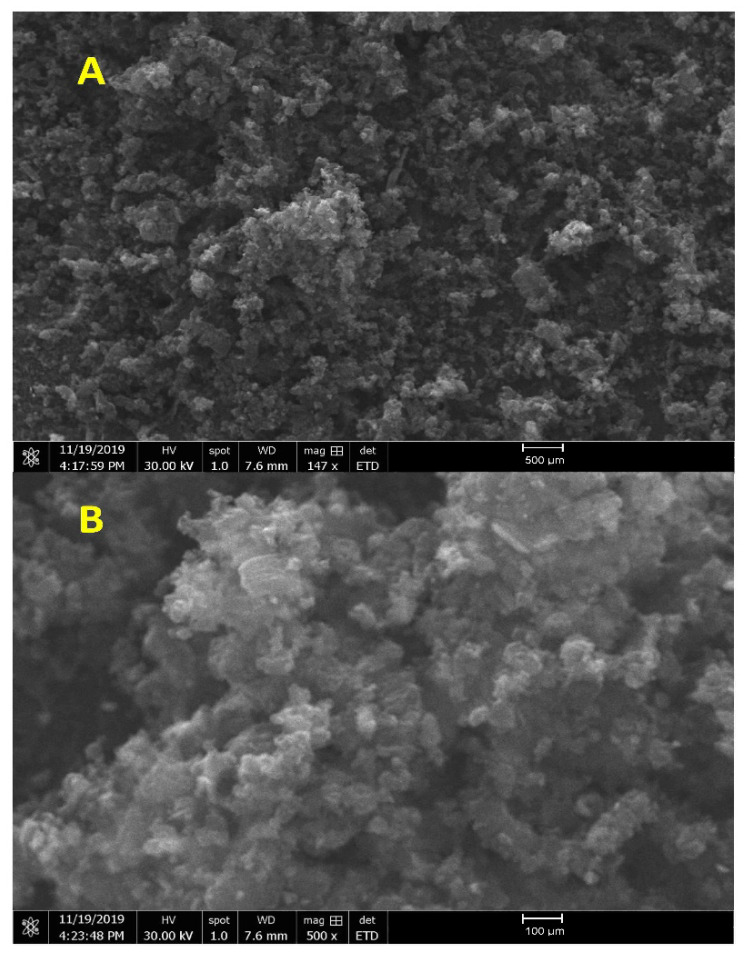
SEM surface analysis of B_4_C with (**A**) 0% graphene, (**B**) 0.2% graphene.

The composite microstructure developed was observed using a scanning electron microscope (SEM). The resulting micrographs confirm a uniform distribution of B_4_C small particles in the base matrix, and it clearly shows no voids or discontinuities. Figure 4a,b show the B_4_C composite and B_4_C–G with 0.2% of graphene content examined under 500 µm magnification, respectively. The effect of grain size was not prominent for the lower concentration of graphene doping, that is, below 0.2% treatment, particularly at the nanoparticles’ surface. For B_4_C composite specimens, at the higher graphene concentrations of 0.4% and 0.6%, the nanoparticle size was observed to be in the range of 30–50 µm. The composite specimens with high graphene concentration showed a surface improvement, as demonstrated in Figure 4a,b. It reduced the particle interconnectivity that would result in high charge transport resistance. Graphene oxide served as a medium to fill the void in pure B_4_C, which led to increased transport properties in the materials.

**Figure 5:**
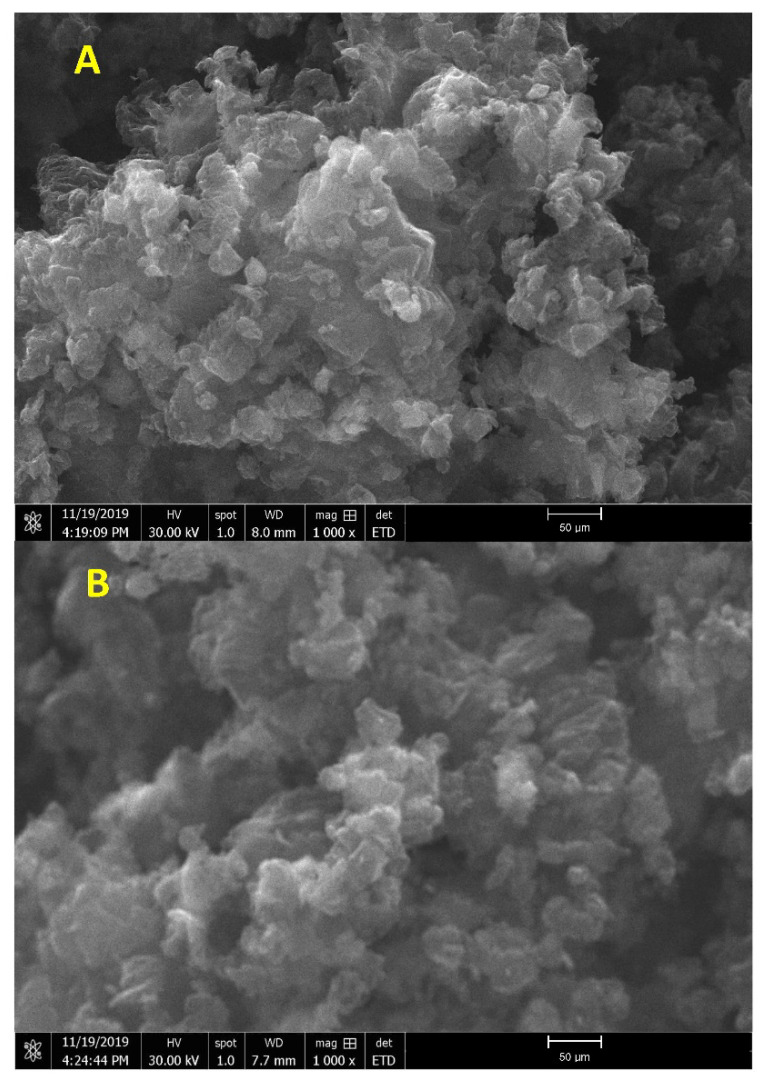
SEM surface analysis of B_4_C with (**A**) 0.4% graphene, (**B**) 0.6% graphene.

The nanoparticles decreased systematically with increasing graphene concentrations, as shown in Figure 5a,b. The B_4_C formed during graphene nanoparticle treatment did not increase B_4_C particle thickness in the morphology in all SEM images with particle size ~30 µm. B_4_C retained its small particle morphology even after undergoing the process. The nanostructure aging enhanced the mechanical properties, as discussed in the mechanical analysis section. The effect of the light from electrons is clearly shown in Figure 5b. 

### 3.2. SEM + EDS Morphology Analysis

**Figure 6:**
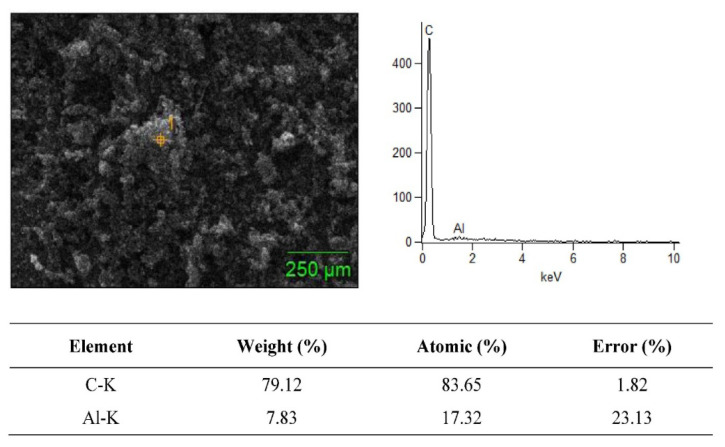
SEM + EDS spectrum of boron carbide + 0% graphene nanoparticles.

**Figure 7:**
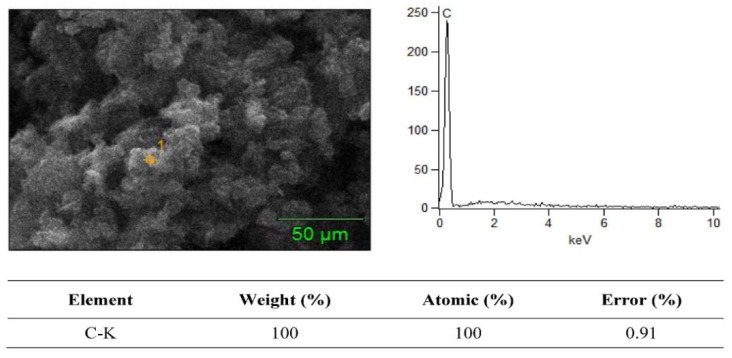
SEM + EDS spectrum of boron carbide + 0.2% graphene nanoparticles.

**Figure 8:**
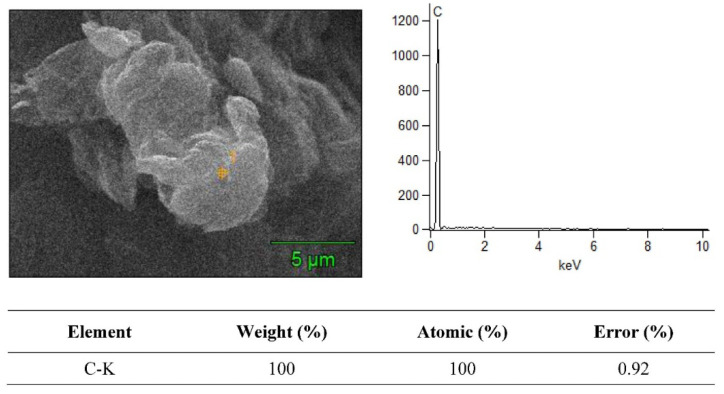
SEM + EDS spectrum of boron carbide + 0.4% graphene nanoparticles.

**Figure 9:**
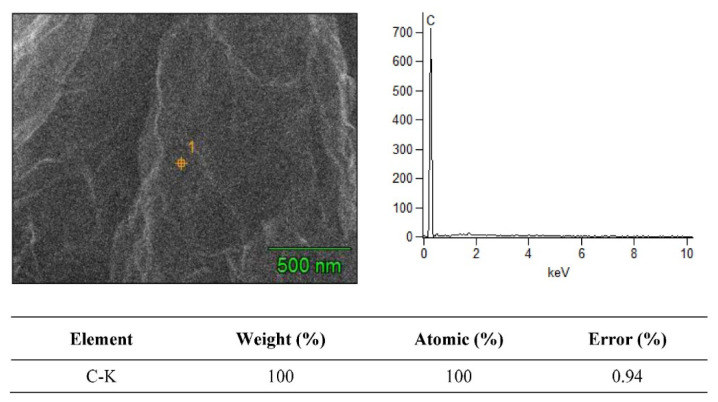
SEM + EDS spectrum of boron carbide + 0.6% graphene nanoparticles.

The SEM merged with EDS analysis is a powerful tool for material characterization. The EDS analyzer is highly useful for many applications due to its ability to identify continuous material shrinking dimensions, and several other elements in composite specimens. In EDS mapping analysis, the amount of penetrated electrons directly relates to the X-ray signals. The SEM + EDS results for the B_4_C–G nanoparticles are illustrated in Figure 6, Figure 7, Figure 8 and Figure 9. The B_4_C + 0% graphene nanoparticle composite specimen was observed to be pure material without treatment in the EDS spectrum. In the observed spectrum, the impurity element weight of C and Al were 79.12% and 7.83%, respectively, as shown in Figure 6. The spectrum values were measured in atomic (%) for C and Al as 83.65 and 17.32, respectively. In Figure 7, Figure 8 and Figure 9, the carbon weight (%) and atomic (%) were the value of 100 for 0.2%, 0.4%, and 0.6% graphene content, respectively, and no EDS signals representing the weightage of other elements was observed. Furthermore, the EDS signals at a voltage of around 0.3 keV manifested by the powder samples are attributed to the C-atom, which is associated with the carbon atoms in the graphene nanoparticles [20]. The EDS signal intensity appeared at a voltage of 0.3 keV, representing the C-atom, which was observed to be higher for the powder sample with a high percentage of graphene content.

As shown in Figure 6, Figure 7, Figure 8 and Figure 9, the EDS observed the elemental mapping, revealing the presence of carbon (C) in the powder samples with 0% graphene, and only C in the specimens reinforced with graphene. EDS analysis has no record of any side products, nor were any contaminations observed at the interface. It was verified that the graphene content, along with the reinforcement, was detected through the EDS. The SEM micrographs with high magnification and EDS spectra revealed a clean and good interface between the matrix (B_4_C) and the graphene component. Boron was not detected for two reasons: the boron has very low, about 0.18 keV, energy at nearly zero, and the EDS analysis scale was not detected. Secondly, due to the heat treatment’s high temperature of 1200 °C, this carbonized all samples, which could have led to boron being hard to detect. 

### 3.3. XRD Analysis 

**Figure 10:**
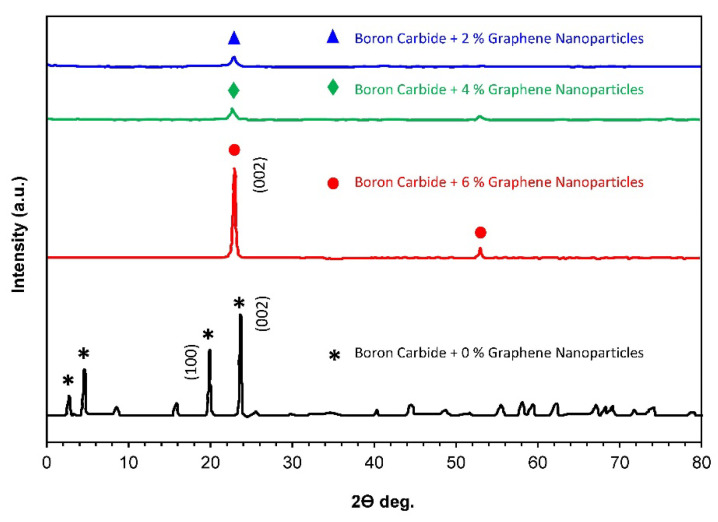
XRD patterns of B_4_C composite material versus 0%, 0.2%, 0.4, and 0.6% graphene nanoparticles.

XRD pattern B_4_C–G composites were detected by utilizing XRD analysis, as shown in Figure 10. The results indicate that the composites were chiefly comprised of graphene nanoparticles (indicated from carbon element peaks) and B_4_C. Graphene nanoparticle peaks were observed at low intensity for the composite specimens, with a low percentage content of graphene. XRD patterns of specimens with 0.2 and 0.4% of graphene content revealed the presence of a characteristic peak for graphene (002) at 22.34° with a d-spacing of 2.78 Å, as shown in Table 2, which usually occurs in the 2θ range of 22° to 27.5° [21]. An increase in the intensity of the graphene’s characteristic peaks for composites with higher graphene loading was observed. A peak at 21.54° with a slight shift from the general 2θ peak range of graphene was observed at a higher percentage of graphene content. The XRD patterns detected for the composite specimen with 0% graphene content were observed to be in good agreement with the reference XRD patterns of the B_4_C phases for the rhombohedral crystal (PDF No. 35-0798) [11]. The basic B_4_C structure can be described in terms of a hexagonal lattice with a (0001) axis, which is in correspondence with the (111) axis in the rhombohedral lattice structure [15]. It dedicated incredibly with 6% graphene, as shown in Figure 10 (002) at 22.34°, but the XRD pattern does not show in all samples due to the overlap.

Table 2 displays the diffraction peaks and respective d-spacing values for all the composite specimens. The d-spacing in the Å units displayed in Table 2 describes the distance in-between the atomic planes that resulted in the rise of diffraction peaks observed for B_4_C and graphene. At a high loading of graphene nanoparticles, the diffraction peak for boron carbide was observed to disappear, and only diffraction peaks representing the presence of graphene were observed. Various other investigators observed carbon atom peaks in the boron carbide matrix [22,23]. Carbon element peaks were detected, and the diffraction structure observed through the analysis may be defined in relation to the diamond lattice having a diffraction plane of (0001) [15].

### 3.4. Raman Analysis

**Figure 11:**
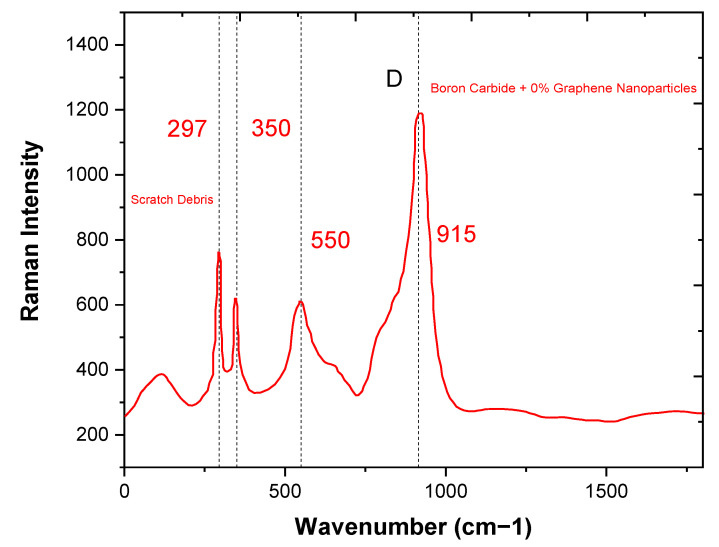
Raman patterns of B_4_C composite material versus 0% graphene nanoparticles.

**Figure 12:**
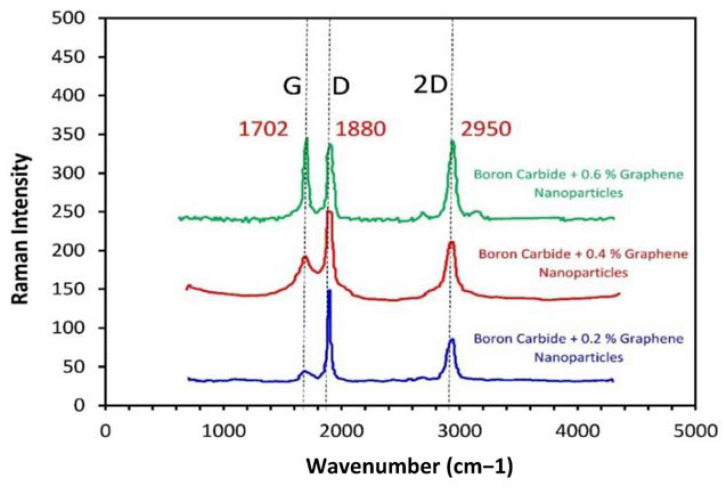
Raman patterns of B_4_C composite material versus 0%, 0.2%, 0.4 and 0.6% graphene nanoparticles.

RAMAN spectroscopy was used in this study to obtain the morphology of the crystals at the microscale level. In this technique, the laser beam is incident on the specimen, which is then reflected back at 180 degrees and is detected by the f/1.5 mm focal length of the collection lens. RAMAN spectra were observed for specimens of boron carbide and graphene composites, with different ratios of graphene as a reinforcement, that is, 0%, 0.2%, 0.4%, and 0.6%. In Figure 11, RAMAN spectra was observed for B_4_C initially, with no graphene content on the sanded specimen in the strain testing where probes were attached at the laboratory. Additionally, in Figure 12, RAMAN spectra was observed for different graphene nanoparticle ratios (0.2%, 0.4% and 0.6%). The RAMAN peaks were observed at nearly 1350 cm^−1^ and 1580 cm^−1,^ and the small peak, was observed at 2700 cm^−1^, indicating that during the sintering process, the graphene structure was not distorted [24]. There was a RAMAN peak shift and broadening in some areas, as shown in Figure 12, due to the coexistence of B_4_C and graphene. The boron carbide crystal lattice consisted of rhombohedral systematic alignment made up of the 12-atom, which are icosahedra, and 3-atom were linear chained as per peaks, as shown in Figure 11 [25]. 

The B_4_C and graphene composite has a perfect rhombohedral crystalline structure. The peaks (at 188, 720, 813, 978, and 1068 cm^−1^) shown in Figure 11 indicate the intra-icosahedral bonding force for the modes of B_4_C, and also the vibrations observed (at 377, 484, and 531 cm^−1^) due to icosahedra. In Figure 12, a small peak can be observed at 2950 cm^−1^, indicating the layered nature of graphene nanoparticles in the B_4_C and graphene nanoparticle composite. A defect in the graphene nanoparticles (GNPs) is indicated at 1702 cm^−1^ by a shoulder near the G band. In the composite, a relatively lower band of G indicates the overlapping of the GNPs. 2-D graphene nanoparticles tend to align perpendicularly when high pressure is applied during sintering. The D/G ratio is so high due to the purity of B_4_C + 0% graphene. The Raman peaks at 1880 cm^−1^ (D band) are essential features of crystalline graphitic structures. The Raman spectrum of B_4_C 0% Graphene nanoparticles can be divided into three ranges: a range with three sharp peaks at 297, 350, and 915 cm^−1^ and a range of 250–1500 cm^−1^ with broad peaks. The Model (Holo-Pro) grating spectrograph disperses the light scattered into several frequency apparatuses and distinguishes them with the (CCD) array detector. In the strain sensing test, the specimen was sanded at locations where the probes were attached. The Raman spectra resulted in the diffraction peak, which could be moved and observed with increasing B_4_C and graphene ratio [13].

### 3.5. FTIR Analysis 

**Figure 13:**
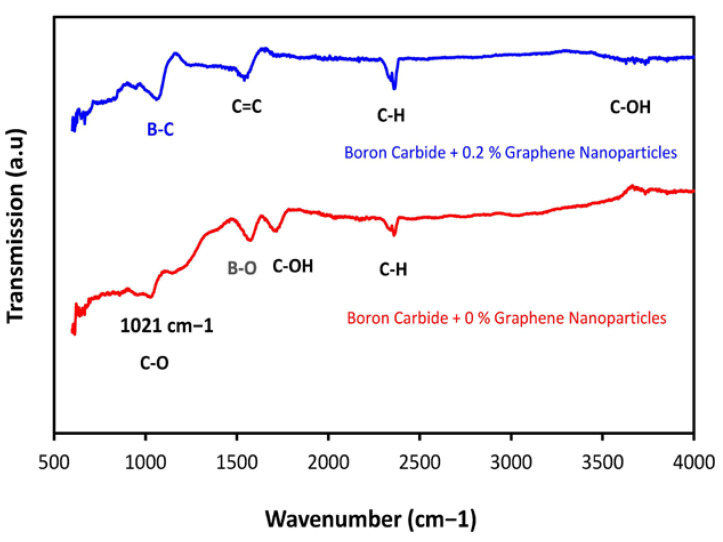
FTIR absorption spectra of the B_4_C + 0% and B_4_C + 0.2% graphene nanoparticles.

**Figure 14:**
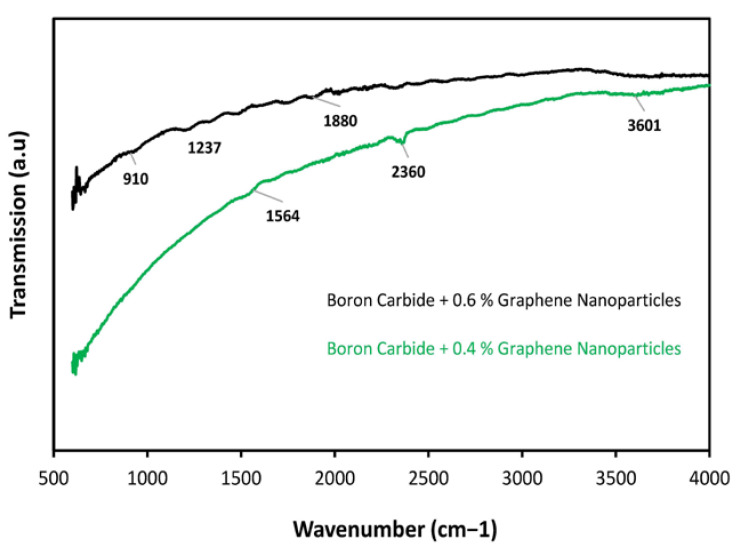
FTIR absorption spectra of the B_4_C + 0.4 graphene nanoparticles and B_4_C + 0.6 graphene nanoparticles.

Fourier transform Infrared spectroscopy was utilized in this research to study the effect of annealing temperature on the chemical interaction of Boron carbide with different ratios of graphene nanoparticles (0%, 0.2%, 0.4%, and 0.6%). A broad FTIR spectrum in the range of 500–4000 cm^−1^ was acquired from FTIR analysis in which the chemical bonding peaks were observed. The peak observed at 1021 cm^−1^ showed the presence of a C-O bond stretching at 0% graphene nanoparticles, as shown in Figure 13. Furthermore, the peaks observed at 2400 cm^−1^ in both curves, that is, for 0% graphene nanoparticles and 0.2% graphene nanoparticles, showed the presence of a C–H bond. However, after calcination with the graphene nanoparticles, an aromatic peak at 1600 cm^−1^ was observed for the C=C bond, which indicated the graphene molecules’ presence and signified the absorption of water molecules. The peak observed at 1043 cm^−1^ was due to the C-O groups stretching [26]. However, it was observed that after increasing the content of graphene nanoparticles, that is, at 0.4% and 0.6%, the oxygen consisted of bonds which were observed to decrease and even disappear, as shown in Figure 14: the upper curve for a composite specimen with 0.6% graphene nanoparticles. In Figure 14, the peak observed at 1237 cm^−1^ is due to the B–C functional group stretching. The presence of a high content of C might have increased the B–C stretching frequency [2].

### 3.6. Tensile Analysis

**Figure 15:**
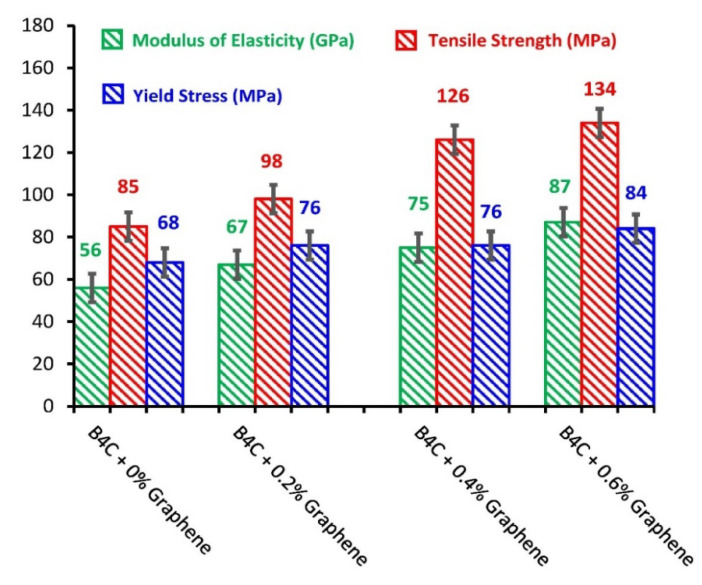
Tensile analysis properties of B_4_C–G composite material.

The mechanical properties of composites are of great significance for their potential use in the application of shielding frames. The impact of the addition of 0.2%, 0.4%, and 0.6% of graphene nanoparticles in B_4_C on the mechanical properties of B_4_C was examined based on the tensile analysis of B_4_C/graphene nanoparticle composites. The tensile properties in terms of (a) yield strength, (b) tensile strength, and (c) modulus of elasticity for graphene nanoparticles reinforced composite specimens, along with the same reading for a composite specimen with 0% graphene nanoparticles content, was ascertained. The tensile analysis demonstrated an increase in tensile strength from 85 MPa to 98 MPa by incorporating only 0.2% of graphene nanoparticle content in the boron carbide matrix, as displayed in Figure 15. The highest value acquired for the highest graphene nanoparticle content (0.6%) composite specimen was observed to be 134 MPa. By construct, the percentage increase in the yield stress value by incorporating a 0.6% graphene nanoparticle was 23.5% from 68 to 84 MPa compared to the specimen with 0% graphene nanoparticles. This prominent increase in tensile strength and yield stress, as shown in Figure 15, was chiefly attributed to the uniform dispersion and distribution of graphene nanoparticles in the boron carbide matrix, as verified by the SEM analysis, and due to the reinforcing nature of graphene. The strong interfacial bonding between graphene nanoparticles and B_4_C also contributed significantly to upgrading the composite shield material’s tensile properties. Furthermore, the uniform dispersion of graphene nanoparticles enabled the transfer of load efficiently from the B_4_C matrix to graphene nanoparticles, and the high mechanical load-bearing ability of graphene nanoparticles increased the overall tensile properties of the composites, based on the ability to add different percentages of graphene nanoparticles, which increased the overall tensile properties of the composites [27,28].

The elastic modulus values in GPa units were also acquired for all the composite specimens, along with yield stress and tensile strength values. A prominent increase in the elastic modulus values was observed by adding graphene nanoparticles in B_4_C, and the elastic modulus values followed an increasing trend with the increase in the loading of graphene nanoparticles. A composite specimen manifested the maximum value of elastic modulus (84 MPa) with 0.6% of graphene nanoparticle content. The elastic modulus results clearly demonstrate the addition of graphene nanoparticles [29]. Retaining their high modulus and strong interfacial interaction with B_4_C, can efficiently and effectively reinforce the B_4_C matrix, which corresponds to the considerable increase in the modulus elasticity of composites. 

### 3.7. Fracture Toughness Analysis

**Figure 16:**
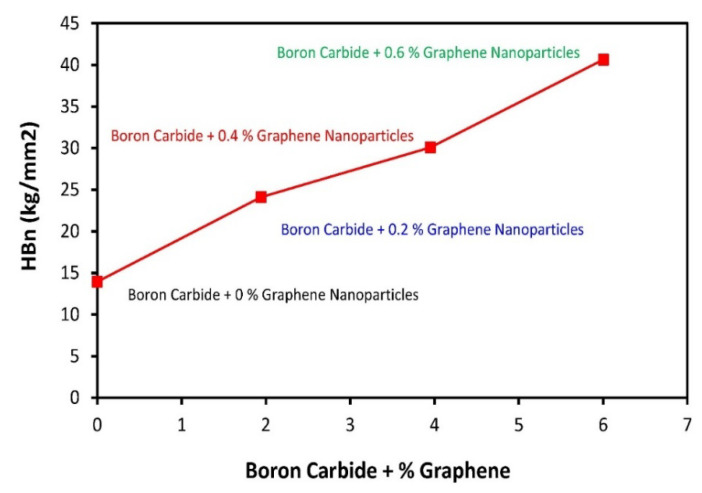
Hardness analysis of B_4_C–G composites material.

Incorporating graphene nanoparticles in the B_4_C had an effect on fracture toughness, which was also investigated. The fracture toughness results shown in Figure 16 reveal a significant increase in the Vickers hardness values of B_4_C–G nanoparticle composites. The fracture toughness values acquired in terms of hardness by the Vickers Indentation Fracture (VIF) test are reported in kg/mm^2^ in Figure 16. When the graphene nanoparticle content reached 0.6%, the composite specimen’s fracture toughness was found to be 42 kg/mm^2^. The composite specimen with 0% graphene content manifested a fracture toughness value of about 14 kg/mm^2^. The value for fracture toughness with the incorporation of 0.2% of graphene nanoparticle content reached 24 kg/mm^2^, and this increase highlights the toughening mechanism of graphene in the boron carbide matrix. The high level of graphene nanoparticle dispersion and distribution in the brittle matrix can absorb the crack propagation energy, and lead to the maximization of the toughening mechanism thanks to graphene’s structural configuration [30].

At high graphene nanoparticle content, that is, 0.4% and 0.6%, a continuous increase in the fracture toughness value was observed. The fracture toughness of all the composites was higher than the composite specimen with no graphene nanoparticles. This is due to the frequent occurrence of crack propagation-toughening mechanisms resulting from the graphene presence with 2D sheets of C-atom in honeycomb configuration [31]. The prominent toughening mechanisms that usually occur due to the presence of graphene are: i-crack bridging, ii-crack branching, and iii-crack deflection. These toughening mechanisms originate from the interaction between graphene nanoparticles and the cracking action [32]. As per the fracture toughness values in this study, the results reveal the effectiveness of graphene nanoparticles in improving boron carbide fracture toughness. 

## 4. Conclusions 

In conclusion, B_4_C–G samples were systematically analyzed in detail using XRD, FTIR, SEM-EDS, Raman spectroscopy, and mechanical analysis. It was found that B_4_C is among the most promising shielding materials and is vitally necessary for novel applications in the shielding industry. By substituting the graphene content in the B_4_C, extraordinary thermal and mechanical stability in the B_4_C–G composite was observed. Moreover, a graphene composition of 6% delivered the highest and strongest tensile strength of 134 MPa and yield strength of 87 MPa. The fracture toughness test also showed an increase in strength from 14 to 42 kg/mm^2^ with increasing doping content from 0% to 6%, respectively. Interestingly, the grain size was observed to decrease with an increase in graphene content in the B_4_C composite. This could have resulted from de-oxidation of the B_4_C–G samples (as shown in FTIR analysis) at 6% graphene content. RAMAN spectroscopic analysis suggests the B_4_C–G composite has a rhombohedral structure, and the highest peaks were observed at 188, 720, 813, 978 and 1068 cm^−1^. The boron has very low, about 0.18 keV, energy—nearly zero. The pristine B_4_C structure studied using RAMAN and XRD analysis has an affirmed rhombohedral structure, which is consistent with previously reported XRD results, and affirms the validity of these findings. B_4_C–G at 6% graphene dopant has shown qualities superior to the B_4_C composite and could be useful as a strong shielding material. B_4_C + %Graphene composites were prepared through fabrication, and under heat treatment, acted as a significantly enhanced shielding frame. Boron carbide has a wide solubility range, but its mechanical and microstructure response effects have shown good results.

## Figures and Tables

**Table 1:** The experimental results for the B_4_C composite material.

Material Composition	B_4_C (Vol.%)	Graphene (Vol.%)	Experimental Mechanical Tests
100–0%	100	0	Tensile, hardness, Indentation Fracture Toughness
99.80–0.2%	99.80	0.2	
99.60–0.4%	99.60	0.4	
99.60–0.6%	99.40	0.6	

**Table 2:** Results of XRD analyses of composites with different ratios of graphene in B_4_C.

Material Composition (B_4_C–G%) (Peak)	2 Ө deg.		d_hk1_ (Å)			
100–0%	5.61	20.32	23.43	0.53	2.46	3.87
99.80–0.2%	-	22.34	-	-	2.78	-
99.60–0.4%	-	22.34	-	-	2.78	-
99.60–0.6%	-	21.54	52.23	-	2.54	4.87

## Data Availability

The data presented in this study are available on request from the corresponding author.

## Sample Availability

Samples of the compounds are not available from the authors.
